# *Umashtanchaeviella plethotricha*, a new genus and species of the family Tetracondylidae (Acari, Oribatida)

**DOI:** 10.3897/zookeys.408.7605

**Published:** 2014-05-12

**Authors:** Sergey G. Ermilov, Alexander E. Anichkin, Andrei V. Tolstikov

**Affiliations:** 1 Tyumen State University, Tyumen, Russia; 2 Joint Russian-Vietnamese Tropical Research and Technological Center, Hanoi-Ho Chi Minh, Vietnam; 3 A.N. Severtsov Institute of Problems of Ecology and Evolution, Russian Academy of Sciences, Moscow, Russia

**Keywords:** Oribatida, Tetracondylidae, new genus, new species, Vietnam

## Abstract

A new genus of oribatid mites of the family Tetracondylidae, *Umashtanchaeviella* **gen. n.**, with type species *Umashtanchaeviella plethotricha* **sp. n.**, is proposed and described from forest litter, the Bu Gia Map National Park, southern Vietnam. The new genus is distinguishable from other otocepheoid genera by the presence of notogastral plethotrichy.^1^

## Introduction

Tetracondylidae Aoki, 1961 (Acari, Oribatida, Otocepheoidea) is the large family of oribatid mites, comprising 24 genera, 3 subgenera, 297 species and 11 subspecies, which are distributed in the Pantropical and Subtropical regions (Subías 2004, online version 2014). The main morphological characters of the subfamily Tetracondylinae were provided by Aoki (1961, 1967). The indentification keys to the many genera and species of tetracondylid mites were presented by Balogh and Balogh (1992, 2002, respectively).

During our studies of the oribatid mite fauna of Bu Gia Map National Park in southern Vietnam, we discovered a species of Tetracondylidae, representing as new genus, *Umashtanchaeviella* gen. n. and *Umashtanchaeviella plethotricha* sp. n.

## Material and methods

Holotype (male) and paratype (male) of *Umashtanchaeviella plethotricha* sp. n. were obtained from southern Vietnam, Binh Phuoc Province, Bu Gia Map National Park, 12°11'N, 107°12'E, 601 m a.s.l., Dipterocarp forest (*Dipterocarpus costatus*), litter (sifting), 13.XI.2013 (collected by A.E. Anichkin and S.G. Ermilov). Specimens are stored in 70% ethanol (omit in tubes).

The soil litter was collected by taking 16 samples using a stainless frame (50 × 50 cm) and passed through a sifter with the mesh size 2 × 2 cm. The fine fraction was placed in a Winkler extractor with a collection bottle containing 100 ml with 75% ethanol. The extractions were conducted at room temperature for more than 20 days.

Holotype and paratype were mounted in lactic acid on temporary cavity slides for measurement and illustration. The body length was measured in lateral view, from the tip of the rostrum to the posterior edge of the ventral plate. The notogastral width refers to the maximum width in dorsal aspect. Lengths of body setae were measured in lateral aspect. All body measurements are presented in micrometers. Formula for leg setation is given in parentheses according to the sequence trochanter–femur–genu–tibia–tarsus (famulus included). Formula for leg solenidia is given in square brackets according to the sequence genu–tibia–tarsus. General terminology used in this paper follows that of Aoki (1965) and Norton and Behan-Pelletier (2009).

## Description of a new genus and a new species

### Family Tetracondylidae

#### Genus Umashtanchaeviella gen. n.

http://zoobank.org/091A7AA9-A6DE-4750-92AF-DB9B9D2887C6

http://species-id.net/wiki/Umashtanchaeviella

##### Diagnosis.

Rostral, lamellar, interlamellar, notogastral and ventral setae setiform. Lateral prodorsal and lateral notogastral condyles of medium size, normally developed. Notogaster with 10 pairs of well developed setae and numerous plethotrichial setae. Ventral neotrichy absent (epimeral formula: 3–1–3–3; anogenital formula: 4–1–2–3). Pedotecta II rectangular. Adanal lyrifissures *iad* located nearly to the anal plates.

##### Type species.

*Umashtanchaeviella plethotricha* sp. n.

##### Etymology.

The specific name is dedicated to our colleague, the acarologist Dr. Umukusum Ya. Shtanchaeva (Universidad Complutense de Madrid, Madrid, Spain), for her extensive contributions to our knowledge of oribatid mites.

##### Remarks.

*Umashtanchaeviella* gen. n. can clearly be distinguished from all the other genera of Otocepheoidea by the following apomorphic character: presence of strong (more 250 pairs) notogastral plethotrichy (versus notogastral plethotrichy absent).

The new genus is most similar to representatives of the genera *Hydroecocepheus* Corpuz-Raros, 1979 (see Corpuz-Raros 1979), *Neotrichocepheus* Hammer, 1973 (see Hammer 1973), *Trichocondyla* J. et P. Balogh, 1986 (see J. et P. Balogh 1986) (all three from Tetracondylidae), and *Megalotocepheus (Archegotocepheus)* Mahunka, 1988 (see Mahunka 1988), *Trichotocepheus* Aoki, 1965 (see Aoki 1965) (both from Otocepheidae) by presence of body neotrichy, however, it can be distinguished from the genera listed above by the localization (notogaster versus ventral side), number (more than 250 pairs versus less than 20 pairs) and morphology (short, thin versus normally developed) of neotrichial setae.

#### Umashtanchaeviella plethotricha sp. n.

http://zoobank.org/7EF674D2-0EAC-4E9F-9AFA-82ACC1782CD8

http://species-id.net/wiki/Umashtanchaeviella_plethotricha

**Figure 1.**
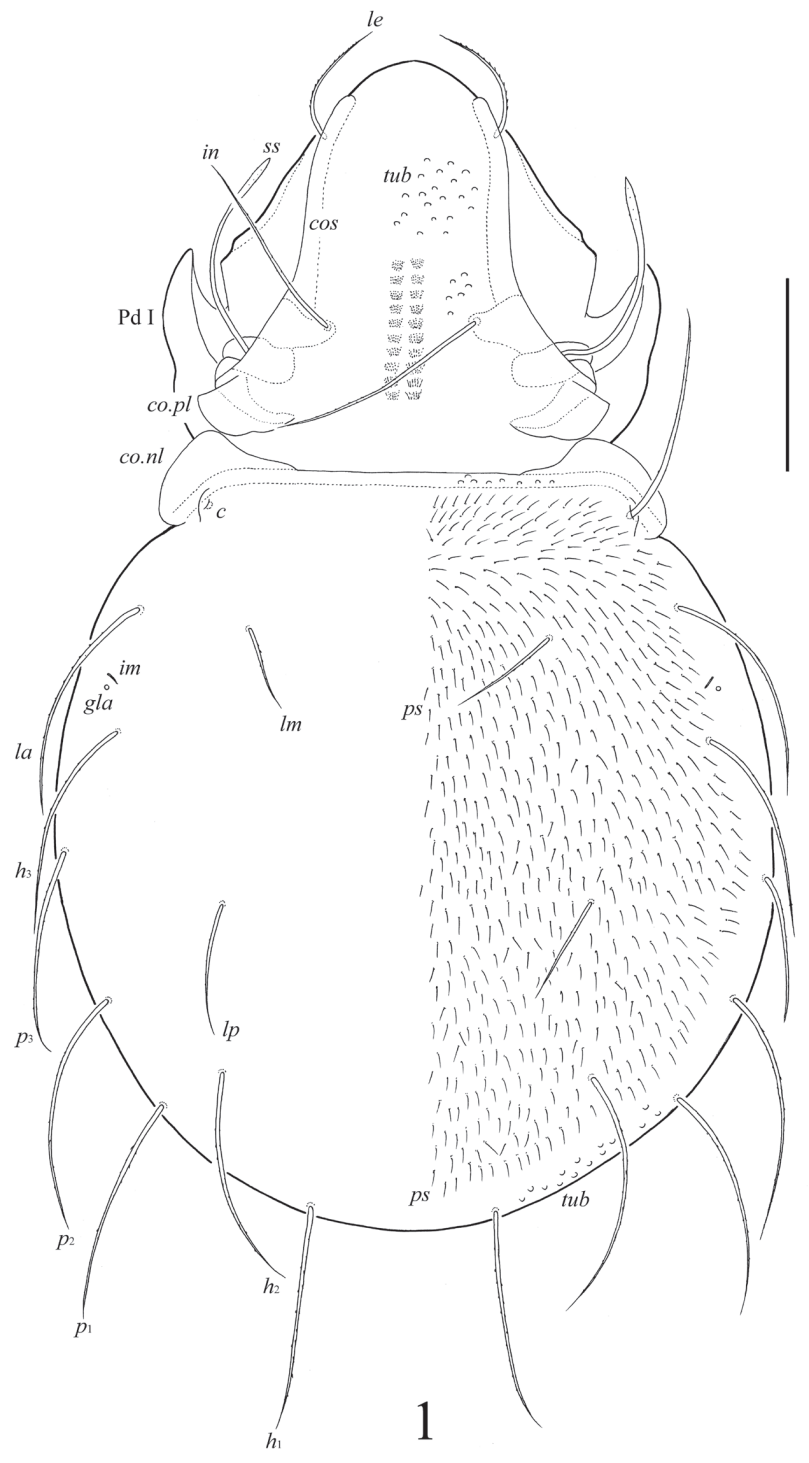
*Umashtanchaeviella plethotricha* sp. n., adult: dorsal view. Scale bar 100 μm.

Figures 1 [Fig F2] [Fig F3] –7

##### Diagnosis.

With character states of *Umashtanchaeviella* gen. n. as listed above. Body surface microfoveolate and microgranulate; surface of dorsal part of prodorsum, lateral sides of notogaster, anterior part of epimere I, anogenital region and anal plates tuberculate. Genital plates striate. Rostral, lamellar and interlamellar setae long, barbed. Bothridial setae with long stalk and short, weakly developed, lanceolate, head. Exobothridial setae short. Medial prodorsal and notogastral condyles absent; lateral prodorsal and notogastral condyles triangular. Ten pairs of notogastral setae setiform, barbed. More than 250 pairs of short, thin plethotrichial setae on notogaster. Epimeral and anogenital setae setiform, barbed. Adanal setae *ad*_1_ shorter than *ad*_2_, *ad*_3_. Lyrifissures *iad* located in paraanal position. Formula for leg setae *u*: L–S–S–S.

##### Description.

*Measurements*. Body length 614 (holotype), 630 (paratype); body width 348 (holotype), 365 (paratype).

*Integument*. Body color light brown. Body surface densely microfoveolate (diameter of foveolae less than 1) and microgranulate (granules elongate, length less than 1). Surface of dorsal part of prodorsum, lateral sides of notogaster, anterior part of epimere I, anogenital region and anal plates tuberculate (*tub*, diameter of tubercles up to 6). Genital plates with thin and numerous stria.

*Prodorsum*. Rostrum widely rounded. Costulae (*cos*) well developed, reaching the insertions of lamellar setae and protruding anteriad. Rostral (*ro*, 77–82), lamellar (*le*, 77–82) and interlamellar (*in*, 155–164) setae setiform, barbed. Bothridial setae (*ss*, 155–164) with long stalk and short, weakly developed, lanceolate, indistinctly barbed head. Exobothridial setae (*ex*) shortest (20), thin, slightly barbed. Medial prodorsal condyles absent. Lateral prodorsal condyles (*co.pl*) triangular, rounded distally.

*Notogaster*. Medial notogastral condyles absent. Lateral notogastral condyles (*co.nl*) large, triangular, rounded distally, connected to lateral prodorsal condyles. Notogaster has a normal complement of typical, identifiable setae, but with an underlying plethotrichy of minute setae. Ten pairs of notogastral setae well developed, setiform, barbed; medial setae *la*, *lm* shorter (73–86) and thinner than other setae (164–176). Distance between setae *h*_1_–*p*_1_ longer than *p*_1_–*p*_2_. Plethotrichial setae (*ps*, more 250 pairs) short (12), thin, straight, smooth, set on small tubercles (insertions of setae visible in high magnification). Lyrifissures *ia*, *im* and opisthonotal gland openings (*gla*) distinct; *ip*, *ih*, *ips* indistinctly developed.

*Gnathosoma*. Morphology of subcapitulum, palps and chelicerae typical for most Tetracondylidae (for example, see Ermilov et al. 2010; Ermilov and Kalúz 2013). Subcapitulum longer than wide (139 × 86). Subcapitular setae setiform, slightly barbed; *a* (20) shorter than *m* and *h* (both 36–41). Adoral setae and their alveoli absent. Palps (86) with setation 0–2–1–3–8(+ω). Solenidion pressed to the palptarsus surface in medio-basal part and distal seta in distal part. Chelicerae (139) with two barbed setae; *cha* (49) longer than *chb* (24). Trägårdh’s organ distinct.

*Epimeral and lateral podosomal regions*. All apodemes (1, 2, sejugal, 3) well visible, Apodemes 4 absent. Epimeral setae setiform, slightly barbed. Setae *1a*, *2a*, *3a*, *4b* shortest (16–20); *4c* (36–41) and *1b*, *1c*, *3b*, *3c* (49) longer; *4a* longest (65–69). Pedotecta I (Pd I) and II (Pd II) well developed. Discidia (*dis*) triangular, pointed anteriorly.

*Anogenital region*. Four pairs of genital (*g*_1_–*g*_4_, 20–24), one pair of aggenital (*ag*, 41–45), three pairs of adanal (*ad*_1_, 65; *ad*_2_, *ad*_3_, 90–94) and two pairs of anal (*an*_1_, *an*_2_, 49–57) setae setiform, barbed. Adanal setae *ad*_1_ located in postanal position, *ad*_2_, *ad*_3_ in adanal position. Distance between setae *ad*_3_–*ad*_3_ longer than *ad*_2_–*ad*_2_ and *ad*_1_–*ad*_1_. Lyrifissures *iad* short, located in paraanal position.

*Legs*. Claw of each tarsus smooth. Tarsi without teeth. Formulae of leg setation and solenidia: I (1–4–3–4–16) [1–2–2], II (1–4–3–3–15) [1–1–2], III (2–3–1–2–15) [1–1–0], IV (1–2–2–2–12) [0–1–0]; homology of setae and solenidia as indicated in Table 1. Morphology of leg segments, setae and solenidia typical for Tetracondylidae (for example see Ermilov et al. 2010; Ermilov and Kalúz 2013). Leg setae *u* setiform (L-type) on tarsi I and thorn-like on tarsi II–IV (S-type).

**Figure 2. F2:**
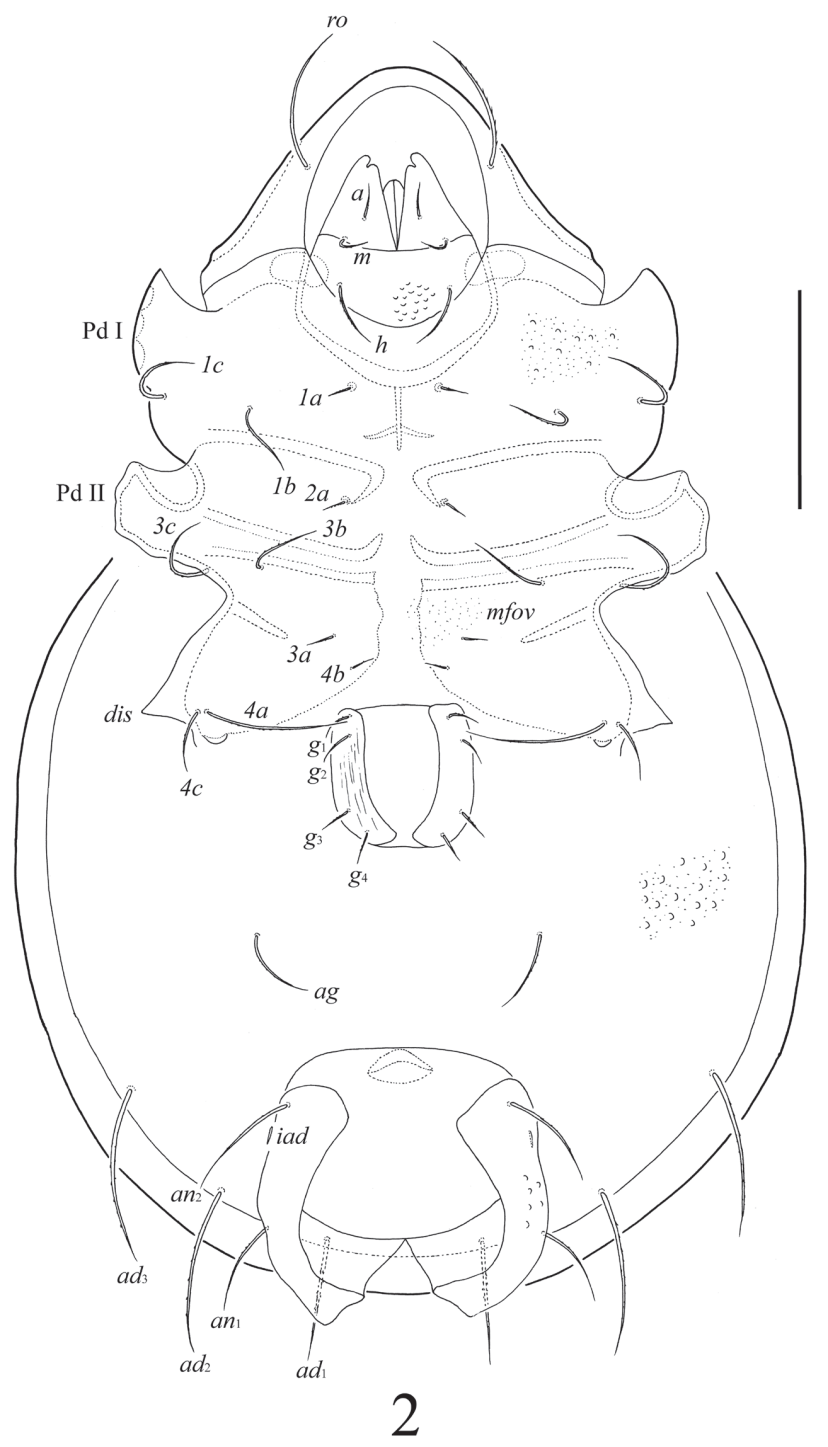
*Umashtanchaeviella plethotricha* sp. n., adult: ventral view (legs not shown). Scale bar 100 μm.

**Figures 3–4. F3:**
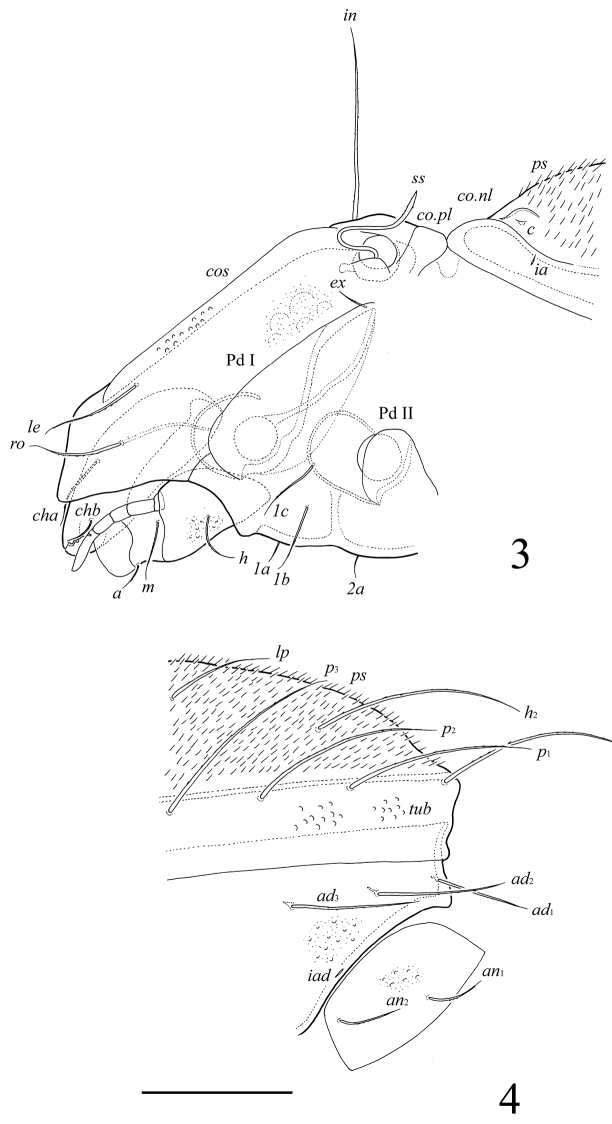
*Umashtanchaeviella plethotricha* sp. n., adult: **3** prodorsum and anterior part of notogaster, lateral view (palp setae not shown) **4** posterior view of notogaster, lateral view. Scale bar 100 μm.

**Figures 5–7.**
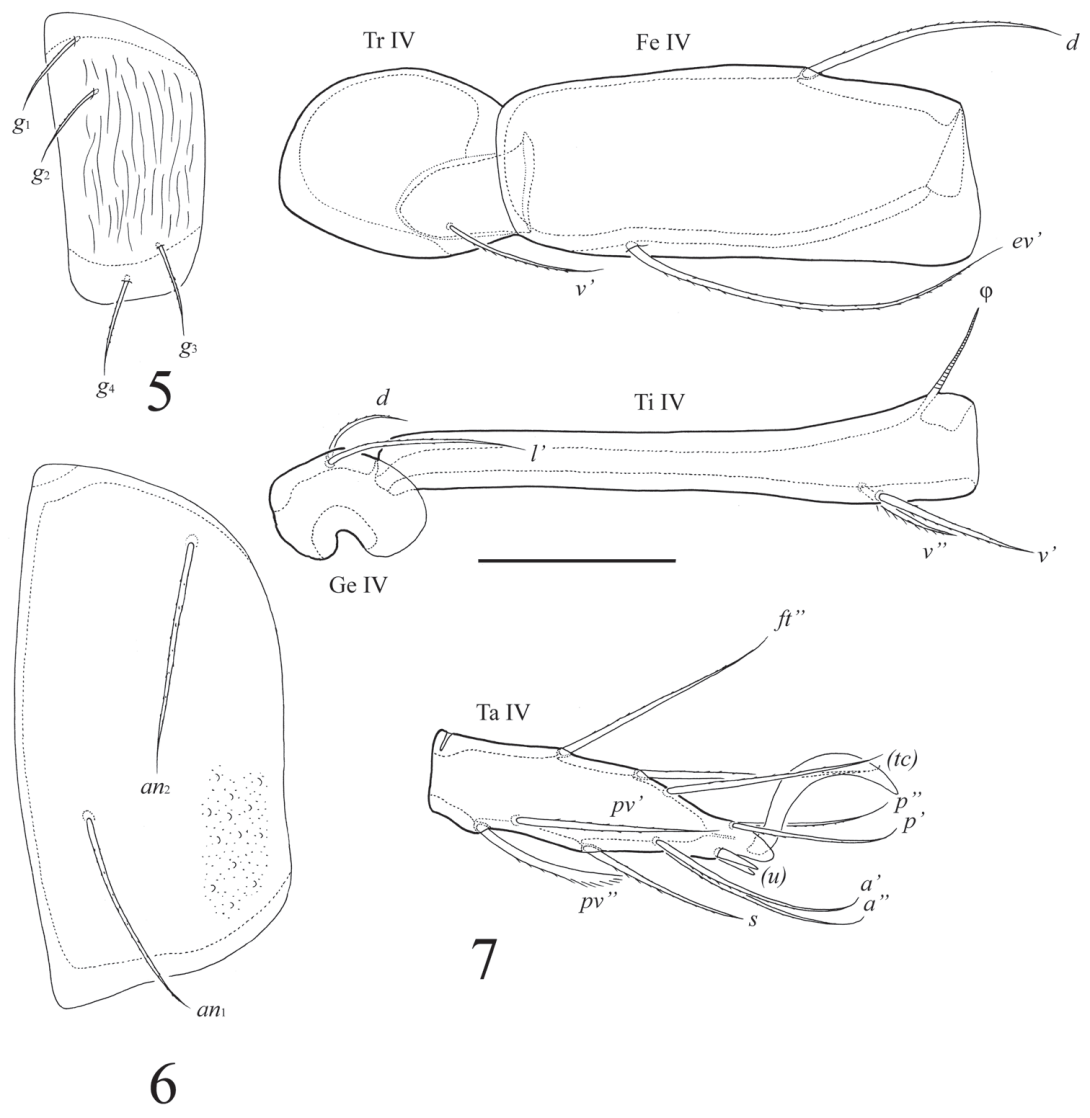
*Umashtanchaeviella plethotricha* sp. n., adult: **5** genital plate, left **6** anal plate, left **7** leg IV, left, antiaxial view. Scale bar 50 μm.

**Table 1.** Leg setation and solenidia of adult *Umashtanchaeviella plethotricha* sp. n.

Leg	Trochanter	Femur	Genu	Tibia	Tarsus
I	*v*’	*d*, *(l)*, *bv*’’	*(l)*, *v*’, σ	*(l)*, *(v)*, φ_1_, φ_2_	*(ft)*, *(tc)*, *(it)*, *(p)*, *(u)*, *(a)*, *s*, *(pv)*, *e*, ω_1_, ω_2_
II	*v*’	*d*, *(l)*, *bv*’’	*(l)*, *v*’, σ	*l*’, *(v)*, φ	*(ft)*, *(tc)*, *(it)*, *(p)*, *(u)*, *(a)*, *s*, *(pv)*, ω_1_, ω_2_
III	*l*’, *v*’	*d*, *l*’, *ev*’	*l*’, σ	*(v)*, φ	*(ft)*, *(tc)*, *(it)*, *(p)*, *(u)*, *(a)*, *s*, *(pv)*
IV	*v*’	*d*, *ev*’	*d*, *l*’	*(v)*, φ	*ft*’’, *(tc)*, *(p)*, *(u)*, *(a)*, *s*, *(pv)*

Roman letters refer to normal setae (*e* to famulus), Greek letters to solenidia. Single prime (’) marks setae on anterior and double prime (’’) setae on posterior side of the given leg segment. Parentheses refer to a pseudosymmetrical pair of setae.

##### Type deposition.

The holotype is deposited in the collection of the Zoological Institute of the Russian Academy of Sciences, St. Petersburg, Russia; one paratype is in the collection of the Tyumen State University Museum of Zoology, Tyumen, Russia.

##### Etymology.

The specific name *plethotricha* refers to the presence of plethotrichial setae on notogaster.

## Supplementary Material

XML Treatment for Umashtanchaeviella

XML Treatment for Umashtanchaeviella plethotricha
